# The taxonomic status of *Dugesia biblica* from Israel and Turkey (Platyhelminthes, Tricladida, Dugesiidae)

**DOI:** 10.3897/zookeys.506.9663

**Published:** 2015-05-28

**Authors:** Eduard Solà, Ronald Sluys, Ori Segev, Leon Blaustein, Marta Riutort

**Affiliations:** 1 Departament de Genètica, Facultat de Biologia and Institut de Recerca de la Biodiversitat (IRBio), Universitat de Barcelona, Av. Diagonal 643, 08028, Barcelona, Spain; 2 Naturalis Biodiversity Center, P.O. Box 9517, 2300 RA Leiden, The Netherlands; 3 Department of Evolutionary & Environmental Biology and Institute of Evolution, Faculty of Sciences, University of Haifa, 3498838, Haifa, Israel

**Keywords:** Platyhelminthes, Tricladida, *Dugesia*, taxonomy, synonymy, biogeography, Israel, *COI*, haplotype, karyology, morphology, Turkey

## Abstract

The taxonomic status of *Dugesia biblica* (Platyhelminthes, Tricladida, Dugesiidae) from Israel and Turkey is problematic due to its morphological similarity with *Dugesia sicula* since these nominal species present overlapping characters. In this study we analyzed histological preparations of specimens of these two nominal species and also compared mitochondrial *COI* gene sequences from Israeli populations to the already known haplotype composition of *Dugesia sicula*. We concluded that these animals belong to the same species and therefore we consider *Dugesia biblica* to be a junior synonym of *Dugesia sicula*. This implies that the distribution range of *Dugesia sicula* is even wider than previously thought, and that the species is present all around the Mediterranean Basin and on many of its islands.

## Introduction

The freshwater planarian fauna of Israel has been relatively well studied (Benazzi and Banchetti 1973; Bromley 1974, 1979, 1980; Bromley and Benazzi 1991). Hitherto, six species of triclad flatworms have been formally described for this country: two species of *Phagocata*, one *Atrioplanaria*, one *Dendrocoelum*, and two *Dugesia* species, most of them inhabiting the northern part of the State (Bromley 1980; Bromley and Benazzi 1991). The two species of *Dugesia* concern *Dugesia golanica* Bromley & Benazzi, 1991 and *Dugesia biblica* Benazzi & Banchetti, 1973. However, so far it has remained uncertain as to whether *Dugesia biblica* is really a species different from *Dugesia sicula* Lepori, 1948 (De Vries 1988).

*Dugesia biblica* was originally described from fissiparous specimens collected from the Jordan River in Israel (Benazzi and Banchetti 1973). Some of these specimens developed a copulatory apparatus under laboratory conditions. Later, Bromley carried out further studies (e.g. karyological and ecological) on this species by analyzing specimens collected from several springs and streams in the Jordan Rift Valley and from the Nahal Qishon water system (Bromley 1974, 1977, 1979). Bromley also found natural sexually reproducing populations (Bromley 1977, 1980). About a decade later, De Vries (1988) described *Dugesia biblica* from two localities in the Mediterranean region of Turkey and noted that the original morphological description of *Dugesia biblica* matches that of *Dugesia sicula*, due to their partially overlapping diagnoses.

In the course of our studies on the evolution and diversification of the genus *Dugesia* in the Mediterranean region (cf. Lázaro et al. 2009; Lázaro and Riutort 2013; Solà et al. 2013; Sluys et al. 2013), we encountered a similar problem when we found many populations throughout Israel to be molecularly identical to *Dugesia sicula*, a species that has never been described from Israel. This induced us to re-evaluate all currently available information. We re-examined the material studied by De Vries (1988) and also specimens from other populations of *Dugesia sicula* that have become available to us over the past few years. Further, we have made extensive samplings throughout Israel in order to determine through DNA sequence analyses and, if possible, by morphological studies, which species are present in the area. On the basis of this integrative approach we were able to evaluate the taxonomic status of nominal *Dugesia biblica*.

## Materials and methods

### Sampling

New samples of *Dugesia* from Israel were obtained during winter, spring and summer seasons in 2009 and 2010. We visited 32 localities (Table 1, Suppl. material 1).

**Table 1.** Israeli sampling localities from where *Dugesia* specimens were collected. The species have been identified on the basis of the *COI* gene sequence.

Code	Locality	Species	Sampling date	Site description	Coordinates
SHE	Ein Shefa	*Dugesia sicula*	06/25/2009	Fast flowing man made spring channel	33°0'34.47"N, 35°8'11.15"E
BAN	Nahal Banias	*Dugesia sicula*	08/27/2009	Fast flowing stream	33°14'47.44"N, 35°41'23.75"E
BET	Nahal Betzet	*Dugesia sicula*	09/01/2009	Isolated temporary pools within dry stream	33°4'32.84"N, 35°13'34.18"E
TEO	Ein Te’o	*Dugesia sicula*	02/03/2010	Shallow spring with moderate water flow	33°7'55.95"N, 35°34'8.54"E
ENU	Ein Nun	*Dugesia sicula*	02/03/2010	Shallow spring with moderate water flow	32°50'18.35"N, 35°30'39.41"E
EHU	Einot Huga	Not *Dugesia sicula*	05/09/2010	Shallow spring - rather saline water ≤2000 mg Cl/l	32°31'2.68"N, 35°32'17.27"E
EOV	Ein Ovdat	*Dugesia sicula*	05/09/2010	Partly connected with slowly flowing spring pools of a desert stream	30°49'25.07"N, 34°45'50.00"E
TZU	Ein Tzuba	*Dugesia sicula*	05/10/2010	Shallow man-made spring pool	31°46'58.33"N, 35°7'45.72"E
SAT	Ein Sataf	*Dugesia sicula*	05/10/2010	Small spring pool inside a man-made underground cave	31°46'15.77"N, 35°7'38.00"E
GED	Ein Gedi	*Dugesia* sp.	08/04/2010	Small shallow spring pool - desert area	31°28'0.60"N, 35°23'19.11"E
DAN	Dan Springs	Not *Dugesia sicula*	08/18/2010	Shallow slowly flowing stream	33°14'56.82"N, 35°39'1.95"E

### DNA extraction and sequencing

Total genomic DNA was extracted by using the commercial reagent DNAzol (Molecular Research Center Inc., Cincinnanti, OH), following the manufacturer’s instructions. A fragment of the cytochrome c oxidase subunit I (*COI*) was amplified using specific primers. Sequences and annealing temperatures for the pair of primers are given in Table 2. Final PCR reaction volume was 25 µl. To 1 µl of DNA sample to amplify we added (1) 5 µl of Promega 5X Buffer, (2) 1 µl of dNTP (10 mM), (3) 0.5 µl of each primer (25 µM), (4) 2 µl of MgCl_2_ (2 mM), (5) 0.15 µl of Taq polymerase (GoTaq® Flexi DNA Polymerase of Promega). Double-distilled and autoclaved water was added to obtain the final PCR volume. The purification of the PCR products was done with the purification kit illustra^TM^ (GFX^TM^ PCR DNA and Gel Band of GE Healthcare) or by using a vacuum system (MultiScreen^TM^_HTS_ Vacuum Manifold of Millipore). Sequencing reactions were performed by using Big-Dye (3.1., Applied Biosystems) with the same primers used to amplify the fragment, or with an inner forward *COI* sequence (COIEF3), due to sequencing problems when using BarT primer. The sequencing reactions were carried out and run in an automated sequencer ABI Prism 3730 by the Unitat de Genòmica of Centres Científics i Tecnològics of the Universitat de Barcelona or by Macrogen Corporation in Europe (Amsterdam, The Netherlands). Obtained chromatograms were visually checked with the software Geneious v. 6.1.7.

**Table 2.** Forward (F) and Reverse (R) primers used in the amplification and sequencing of the *COI* mitochondrial gene sequence.

Name	Direction	Sequence 5’−3’	Annealing temperature (°C)	Source
BarT	F	ATGACDGCSCATGGTTTAATAATGAT	43	Álvarez-Presas et al. 2011
COIEF3	F	CCWCGTGCWAATAATTTRAG	43	Solà et al. 2013
COIR	R	CCWGTYARMCCHCCWAYAGTAAA	43	Lázaro et al. 2009

### Alignment and haplotype network

The number of *Dugesia* individuals analyzed per locality ranged between 1 and 7, depending on the available number of specimens and the success of sequencing (Table 3). The sequences were aligned online with MAFFT version 7 by setting the iterative refinement method in G-INS-i (Katoh and Standley 2013). We used the software Network version 4.613 (Bandelt et al. 1999), using Median-Joining for network calculations. Parameters were set as default.

**Table 3.** Details on the Israeli individuals sequenced for the present work.

Individual	Locality	Polymorphic	Haplogroup	Haplotype in Figure 2	GenBank Acc. Number
D01TEO	Ein Te’o	No	A	7	KR140038
D01BAN	Nahal Banias	No	B	2	KR140035
D02BAN		Yes	−	−	KR140040
D03BAN		Yes	−	−	KR140045
D04BAN		No	B	2	KR140049
D02SHE	Ein Sheva	No	B	3	KR140043
D03SHE		No	B	3	KR140047
D04SHE		Yes	−	−	KR140052
D05SHE		Yes	−	−	KR140056
D06SHE		No	B	3	KR140059
D01BET	Nahal Betzet	No	B	8	KR140036
D02BET		No	B	8	KR140041
D03BET		No	B	8	KR140046
D04BET		No	B	3	KR140050
D05BET		No	B	3	KR140053
D01TZU	Ein Tzuba	No	B	4	KR140039
D02TZU		No	B	4	KR140044
D03TZU		No	B	4	KR140048
D07TZU		No	B	4	KR140062
D08TZU		No	B	4	KR140063
D09TZU		No	B	4	KR140066
D10TZU		No	B	4	KR140067
D04SAT	Ein Sataf	No	B	5	KR140051
D05SAT		No	B	5	KR140055
D06SAT		No	B	5	KR140058
D07SAT		No	B	5	KR140061
D11SAT		Yes	−	−	KR140068
D06EOV	Ein Ovdat	No	B	1	KR140057
D07EOV		No	B	1	KR140060
D09EOV		No	B	6	KR140065
D01ENU	Ein Nun	Yes	−	−	KR140037
D02ENU		Yes	−	−	KR140042
D05ENU		Yes	−	−	KR140054
D09ENU		Yes	−	−	KR140064
D16ENU		Yes	−	−	KR140069

### Preparations

Material examined (collections Naturalis Biodiversity Center, Leiden):

*Dugesia biblica*:

ZMA V.Pl. 698.1, Banias Waterfall, Israel, transverse sections on 6 slides, V.Pl. 698.2, ibid., sagittal sections on 8 slides.

ZMA V.Pl. 699.1, Ein El Hanea, Israel, January 1972, sagittal sections on 8 slides; V.Pl. 699.2., ibid., transverse sections on 12 slides.

ZMA V. Pl. 813.1, spring, 5 km NW of Bucak, Turkey, sagittal sections on 2 slides; V.Pl. 813.2, ibid., sagittal sections on 3 slides; V.Pl. 813.3, ibid., frontal sections on 2 slides.

ZMA V.Pl. 814.1, stream near Yerkopru, Hadim, Turkey, sagittal sections on 4 slides; V.Pl. 814.2, ibid., sagittal sections on 3 slides; V.Pl. 814.3, ibid., frontal sections on 3 slides.

*Dugesia sicula*:

ZMA V.Pl. 7152.1, Tripes, Chios, Greece, 2 May 2010, sagittal sections on 10 slides.

## Results

### Samples

**Figure 1.**
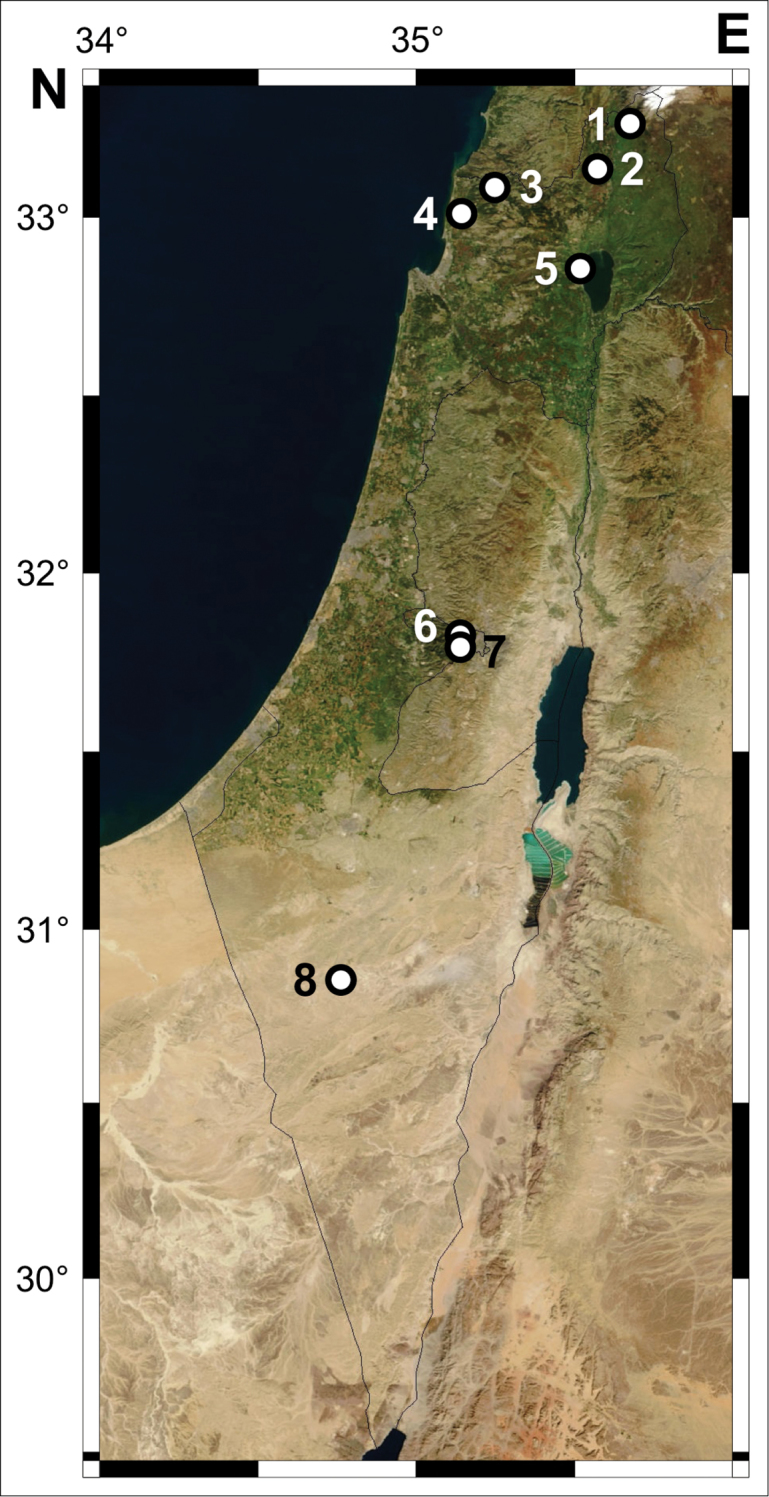
Map of Israeli localities sampled for this study: **1** Nahal Banias **2** Ein Te’o **3** Nahal Betzet **4** Ein Shefa **5** Ein Nun **6** Ein Tzuba **7** Ein Sataf **8** Ein Ovdat. For locality details, see Table 1.

Out of the 32 localities that we visited in Israel, about one-third (11) yielded specimens of *Dugesia* (Fig. 1, Table 1, Suppl. material 1). At two of these localities we found some *Dugesia* specimens that were molecularly different from *Dugesia biblica* or *Dugesia sicula*. One of these two populations, from Dan Springs (Table 1), might be *Dugesia golanica*, which was originally described from Dan Springs and also from Banyas Springs, in the vicinity of Dan Springs. Our second series of specimens, from Einot Huga, may represent a different species, according to its very distant phylogenetic position (data not shown). Perhaps specimens from the latter locality represent *Dugesia salina* (Whitehouse, 1913), currently a *species inquirenda*. According to Bromley (1980), the chromosomal complement for *Dugesia salina* is 2n = 16 and is different from *Dugesia golanica*, although she did not describe the chromosomes from the latter species. Whitehouse (1913) reported *Dugesia salina* from near et-Tabghah (= En Sheva), while Bromley (1974, 1980) reported populations from En Sheva, En Soda, and from River Jordan at its outlet from Lake Kinneret. Our locality of Einot Huga is actually very close to En Soda. However, as these two species, *Dugesia golanica* and *Dugesia salina*, fall outside of the scope of the present study, we did not include the specimens in our analyses.

Unfortunately, preservation and histological problems eventually prevented us of carrying out detailed morphological analyses on the reproductive apparatus of Israeli *Dugesia* specimens from the various newly sampled populations (Table 1, specimens from localities EOV, EHU, TZU, DAN).

### Alignment and haplotype networks

We were successful in obtaining *COI* sequences for 8 out of the 9 sampling localities; 25 out of the 35 sequences obtained for the present study presented no polymorphism, while the remaining sequences showed between 1 and 12 polymorphic positions. We used both the 25 *COI* non-polymorphic sequences from presumed Israeli *Dugesia biblica* obtained for this study (Table 3), as well as those of *Dugesia sicula*, as obtained in a previous phylogeographic study of this species (95 sequences; GenBank Acc. number: KC536630–KC536644 and KC577271–KC577350; Lázaro and Riutort 2013) in order to carry out a haplotype network analysis. The alignment contained 120 *COI* sequences, included 604 nucleotides, and presented 15 polymorphic positions.

**Figure 2.**
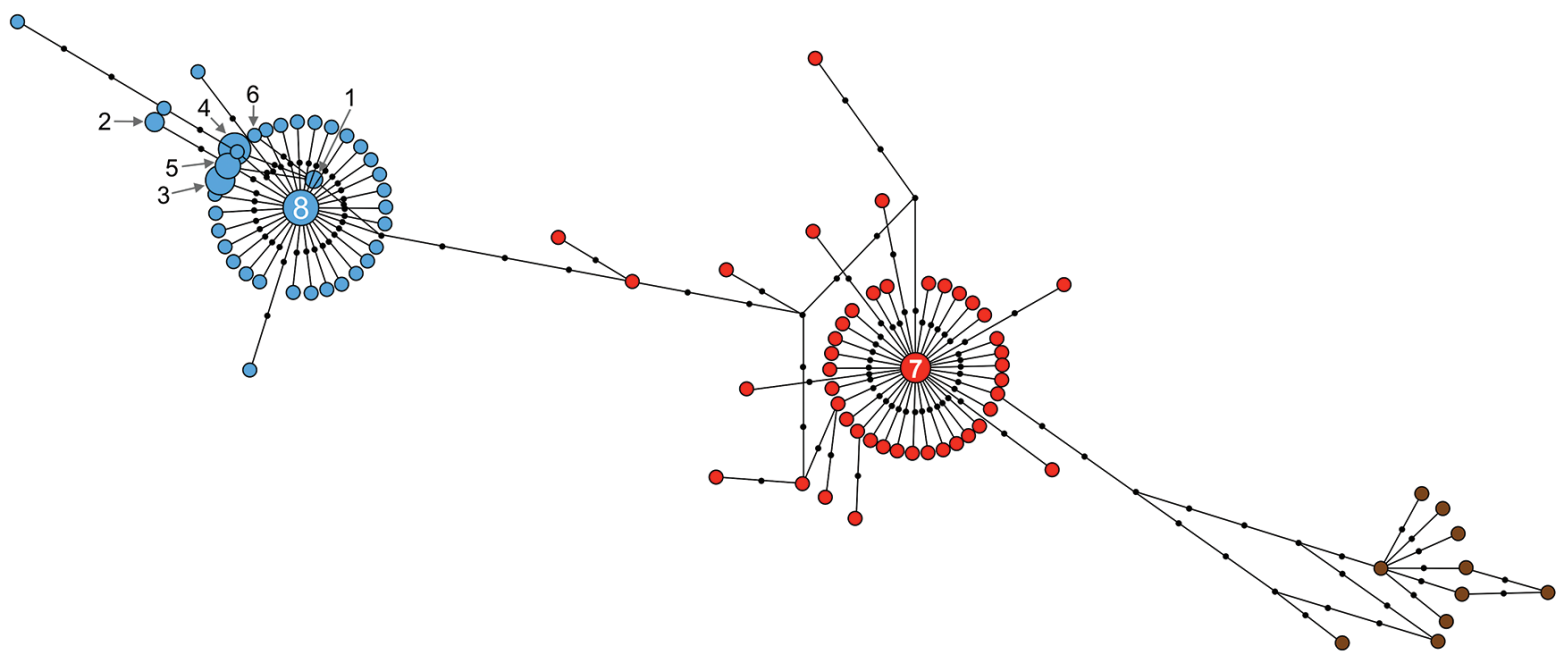
Haplotype network of *Dugesia sicula* and presumed *Dugesia biblica* *COI* sequences. Filled red circles correspond to haplogroup **A**, filled blue circles correspond to haplogroup **B**, and filled brown circles correspond to haplogroup **C** of *Dugesia sicula* (as defined in Lázaro and Riutort 2013). The size of the coloured circles is proportional to the haplotype representation. Small black dots indicate intermediate haplotypes (not-obtained). Numbers indicate the identity of Israeli haplotypes; for further details see Table 3.

Most of the Israeli *COI* haplotypes are identical or are only 1−4 positions removed from the major *Dugesia sicula* *COI* haplotype B (Fig. 2). One individual sequence (D01TEO) belongs to the other major *COI* haplotype, viz haplotype A (cf. Lázaro and Riutort 2013; Fig. 2). The geographical extension of the B haplogroup in the present study widens its known distributional range to the coast of Israel. The A haplogroup ranges from Morocco to Israel on both sides of the Mediterranean Sea.

Additionally, we compared the polymorphic sequences of the Israeli *Dugesia* not included in the haplotype network (Table 3) with the sequences of *Dugesia sicula* *COI* haplotypes already defined (Lázaro and Riutort 2013; present work). We found that the polymorphic positions corresponded with those that are variable between haplotypes, indicating that these organisms were heteroplasmic for various known haplotypes.

The results of our molecular analyses suggest a wide distribution of *Dugesia sicula* throughout Israel (Fig. 1), as well as the absence of any other molecularly related species in this area.

### Morphological and karyological comparison between *Dugesia biblica* and *Dugesia sicula*

We have been unable to find any stable structural morphological difference between *sicula* populations and presumed *biblica* populations. All of these animals are characterized by distinctly acentral opening of the ejaculatory duct; asymmetrical oviducal openings into the bursal canal; rather thick layer of circular muscles around bursal canal; bursal canal that runs somewhat laterally to the penis; zone of mesenchymatic gland cells around bursal canal; somewhat bilobed seminal vesicle; somewhat irregularly running bursal canal, with irregular diameter; distinct patch of cyanophil secretion in dorsal section of penis papilla. Benazzi and Banchetti (1973) described for *Dugesia biblica* an outer pharynx musculature consisting of three layers. However, De Vries (1988) already correctly observed that such an extra, third layer is not present in *biblica* specimens from Israel. Bromley (1979) described atrial folds for *Dugesia biblica*, but such structures were not observed by us in the available material from Israel. The vacuolated tissue that Bromley (1979) described for the penis of *Dugesia biblica* in our opinion merely concerns tears in the mesenchyme of the penis papilla. Such tears or spaces in the dorsal part of the penis papilla, near its tip, were observed in histological preparations of specimens from several populations of *Dugesia sicula*, e.g. specimen ZMA V.Pl. 7152.1 from Chios.

Characteristic of *Dugesia biblica* is the occurrence in the field of a sexually reproducing diploid form with a chromosome complement of 2n = 18, and a triploid form that reproduces asexually by fission with a set of 3n = 27 + 1−5 supernumerary chromosomes. Under laboratory conditions, the normally fissioning animals can be induced to develop reproductive organs. The structure of the copulatory organs of these sexualized animals is identical to that of the normally sexually reproducing diploid forms. However, in the diploid forms, testes and ovaries show their normal dimensions and development, whereas in the sexualized animals the testes are underdeveloped and the ovaries hyperplasic (cf. Bromley 1974, 1977, 1979). The difference in karyology between the asexual individuals and the naturally sexual animals induced Bromley (1979, 1980) to coin the subspecies *Dugesia biblica biblica* Benazzi & Banchetti, 1973 and *Dugesia biblica monticola* Bromley, 1980, respectively.

The situation that (1) in the field some populations may reproduce asexually and show a triploid set of 3n = 27 + 2−3 B chromosomes, (2) others reproduce sexually and show a complement of 2n = 18 gradually decreasing, metacentric chromosomes, and (3) sexualized, triploid specimens show hyperplasic ovaries and poorly developed testes is well-known for *Dugesia sicula* (cf. Charni et al. 2004 and references therein). Thus, also from this perspective, there seems to be no difference between *Dugesia sicula* and *Dugesia biblica*.

## Conclusion: the taxonomic status of *Dugesia biblica*

In addition to the morphological and karyological similarities between nominal *Dugesia biblica* and *Dugesia sicula* (see above), our molecular analysis shows presumed *biblica* populations to be molecularly indistinguishable from *sicula* populations. The Israeli haplotypes obtained are either identical to previously obtained *sicula* or present few differences from these. Therefore, on the basis of our integrative analysis, we consider *Dugesia biblica* to be a junior synonym of *Dugesia sicula*.

**Figure 3.**
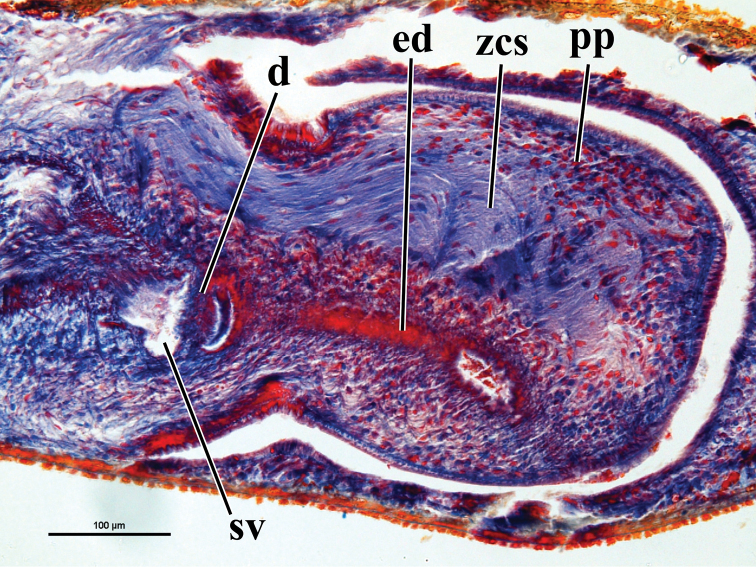
Presumed *Dugesia sicula* from Bucak, Turkey (ZMA V.Pl. 813.2), showing the presence of the zone of cyanophil secretion in the penis papilla. Abbreviations: **d** diaphragm **ed** ejaculatory duct **pp** penis papilla **sv** seminal vesicle **zcs** zone of cyanophil secretion.

This conclusion holds true for one of the two Turkish populations of presumed *biblica* described by De Vries (1988), viz. ZMA V.Pl. 814 from Yerkopru. But the other population (ZMA V.Pl. 813 from 5 km NW of Bucak) concerns animals that are morphologically somewhat different from *Dugesia sicula*. Foremost, the ejaculatory duct does not have a subterminal opening (cf. De Vries 1988, Fig. 2). Other differences concern the position of the ovaries at 1/3^rd^ – 1/4^th^ of the distance between the brain and the root of the pharynx (1/4^th^ – 1/5^th^ in *Dugesia sicula*), the much wider bursal canal, which is surrounded by a much thinner layer of circular muscle (depicted far too thick in De Vries 1988, Fig. 2), and the smaller copulatory bursa in the specimens from Bucak. The animals from Bucak agree with *Dugesia sicula* in the presence of numerous mesenchymal glands discharging their erythrophil secretion into the lining epithelium of the bursal canal, the presence of the zone of cyanophil secretion in the penis papilla (Fig. 3), and the asymmetrical openings of the oviducts into the bursal canal. In several respects the animals from Bucak remind one of *Dugesia naiadis* Sluys, 2013 from Chios, albeit that in the latter the oviducts open symmetrically into the bursal canal, in contrast to the asymmetrical oviducal openings in the Bucak specimens (cf. De Vries 1988, Fig. 2). However, for the moment we refrain from assigning the animals from Bucak to a different and possibly new species of *Dugesia* and postpone any taxonomic decision until more material has become available for both morphological and molecular analyses.

## Supplementary materials

Supplementary material 1 Supplementary Table 1 Data type: occurence Explanation note: Localities in Israel from which no specimens of *Dugesia* could be obtained during our samplings. This dataset is made available under the Open Database License (http://opendatacommons.org/licenses/odbl/1.0/). The Open Database License (ODbL) is a license agreement intended to allow users to freely share, modify, and use this Dataset while maintaining this same freedom for others, provided that the original source and author(s) are credited.Eduard Solà, Ronald Sluys, Ori Segev, Leon Blaustein, Marta Riutort
